# A Real-World Study on Pulmonary Arterial Hypertension in Bulgaria: A Single-Center Retrospective Study From 2012 to 2022

**DOI:** 10.1155/crp/5579064

**Published:** 2025-05-20

**Authors:** Vasil Velchev, Arman Postadzhiyan, Sarkis Kalustian, Simona Markova, Mihaela Manolova, Damyan Boychev, Daniel Penchev, Martina Nacheva, Elina Petrova

**Affiliations:** ^1^Cardiology Clinic, UMBAL “Saint Anna” – Sofia, Sofia, Bulgaria; ^2^Data Science, Sqilline, Sofia, Bulgaria; ^3^Market Access, MSD Bulgaria, Sofia, Bulgaria

**Keywords:** compliance, epidemiology, monotherapy, PAH, pulmonary hypertension, survival, treatment

## Abstract

**Purpose:** This study aimed to analyze the epidemiological, clinical, and therapeutic characteristics of patients with pulmonary arterial hypertension (PAH) treated at a major reference center in Bulgaria and to assess treatment patterns, patient compliance, and overall survival.

**Principal Results:** The epidemiological data revealed that 69.5% of the patients were female, with a mean age of 52 years. The majority of patients were diagnosed at advanced stages of PAH, with 92.1% classified as World Health Organization Functional Class III. Monotherapy was the most common treatment regimen, used by 61.4% of patients, despite advanced disease. Patients who adhered to treatment demonstrated significantly longer overall survival (78.9 months) compared to those lost to follow-up (50.8 months). The study also identified a 31% rate of noncompliance, with patients missing follow-up visits and becoming ineligible for further therapy.

**Major Conclusions:** The findings highlight the need for earlier diagnosis and more aggressive treatment strategies, as monotherapy appears insufficient for optimal outcomes in advanced PAH. Establishing a national PAH registry and increasing disease awareness could facilitate earlier interventions and improve patient outcomes in Bulgaria.

**Summary**

• The majority of PAH patients in Bulgaria present with advanced disease at diagnosis.
• Monotherapy is widely used despite ERS/ESC guidelines recommending combination therapy.
• Treatment compliance significantly improves overall survival in PAH patients.
• A national PAH registry could improve diagnosis and treatment strategies in Bulgaria.

## 1. Introduction

Pulmonary arterial hypertension (PAH) is a rare and progressive disease characterized by pathological remodeling and narrowing of the pulmonary arteries. This narrowing increases pulmonary vascular resistance (PVR) and pulmonary arterial pressure, which, if untreated, results in right ventricular failure and death [1]. PAH is hemodynamically defined by a mean pulmonary arterial pressure (mPAP) > 20 mmHg, a pulmonary capillary wedge pressure (PCWP) ≤ 15 mmHg, and a PVR ≥ 2 Wood units (WUs) [2].

Registry data from developed countries report a PAH incidence of approximately 6 cases per million adults annually, with a prevalence of 48–55 cases per million [2, 3]. However, the incidence and prevalence of PAH in Bulgaria remain unknown, largely due to the absence of a national PAH registry. The nonspecific symptoms of PAH, including dyspnea, fatigue, angina, and syncope, often lead to delayed diagnosis. Definitive diagnosis requires right heart catheterization (RHC), performed at specialized reference centers [4]. Currently, Bulgaria has three such centers, two of which are located in the capital, Sofia. Delayed diagnosis remains a significant challenge in Bulgaria, contributing to poorer outcomes and increased mortality [5–7].

PAH management in Bulgaria is complicated by limited access to advanced therapies. Patients must visit their reference centers regularly to maintain access to specific oral treatment regimens, as parenteral therapies are not reimbursed. The absence of a national PAH registry further complicates efforts to systematically collect and assess data on the clinical burden and healthcare needs of this population. This data gap hinders a full understanding of PAH epidemiology in Bulgaria and limits the implementation of targeted healthcare strategies.

This study aims to provide a detailed analysis of the epidemiological, clinical, and therapeutic landscape of PAH in Bulgaria, using retrospective data from a cohort of 194 patients treated at one of the country's primary PAH reference centers from 2012 to 2022. By examining demographic characteristics, diagnostic indicators (including RHC, imaging, hemodynamics, exercise capacity, and biomarkers), treatment regimens, and survival outcomes, this study seeks to offer a comprehensive overview of PAH management in Bulgaria.

## 2. Materials and Methods

This retrospective study utilized health records from St. Anna University Hospital in Sofia, Bulgaria, the largest treatment center for PAH in the country. The study included all adult patients diagnosed with PAH between January 2012 and December 2022. All available patient records from this period were included in the analysis to ensure a comprehensive overview of the patient cohort, with no exclusion criteria applied.

### 2.1. Patient Records and Data Collection

Patient records consisted of epicrises (discharge summaries) issued during hospitalizations, conducted either annually or biannually. These epicrises contained demographic information (gender and age), clinical data, diagnostic parameters, and treatment regimens. The primary clinical indicators extracted from the records included RHC results, hemodynamic parameters (cardiac output [CO] and mPAP), functional status (measured via the World Health Organization Functional Class [WHO-FC]), exercise capacity (assessed by the 6 min walk distance [6MWD]), and biomarker levels (brain natriuretic peptide [BNP] and N-terminal proBNP [NT-proBNP]). Data from the first patient visit were used as a baseline for further analyses.

In addition to the clinical and diagnostic data, death status was collected to estimate overall survival (OS). Mortality data were obtained from the Civil Registration and Administration Services General Directorate (CRASGD), the official national registry for vital statistics in Bulgaria.

### 2.2. Treatment Compliance

Patient compliance with PAH–specific treatment regimens was assessed. Treatment compliance was evaluated based on the patient's adherence to scheduled follow-up visits. A patient was deemed compliant if their follow-up visit occurred within 7 months of the previous visit. Noncompliant patients, labeled as lost to follow-up, were defined as those who did not return for scheduled visits within this timeframe. Following Bulgaria's reimbursement policies, patients who missed follow-up visits were unable to continue receiving specific PAH therapies, resulting in the effective discontinuation of treatment.

To monitor compliance over time, patient data from 2023 were also analyzed, determining whether individuals in the cohort continued to adhere to treatment regimens. Patients who returned for follow-up in 2023 were categorized as compliant, while those who did not were classified as lost to follow-up.

### 2.3. Statistical Analysis

Baseline characteristics of the patient cohort were summarized using descriptive statistics. Mean, standard deviation, median, and counts were reported for continuous variables, while categorical data were presented as frequencies and percentages. Kaplan–Meier survival analysis was conducted to estimate OS from the date of PAH diagnosis to the date of death or the end of the study period.

To explore potential associations between survival outcomes and clinical characteristics, correlations between death status and variables such as WHO-FC, gender, age, PAH subtype, CO, mPAP, and 6MWD at the time of the first visit were assessed. Statistical significance was assessed using appropriate tests, with a *p* value < 0.05 considered significant.

### 2.4. Ethical Considerations

This study was conducted following the principles outlined in the Declaration of Helsinki. All data were anonymized to protect patient confidentiality, and the study protocol was approved by the Institutional Review Board (IRB) of St. Anna University Hospital.

## 3. Results

Table 1 summarizes the baseline characteristics of the 194 PAH patients included in the study. The majority of the patients presented with advanced disease, with 92.1% classified as WHO-FC III at diagnosis. Hemodynamic parameters obtained from RHC, including mPAP, right atrial pressure (RAp), CO, and PVR, indicated severe circulatory impairment at the initial visit. These findings aligned with other clinical measures, such as echocardiographic parameters, the 6MWD, and biomarker levels (BNP and NT-proBNP), further confirming the advanced disease stage at diagnosis.

### 3.1. Comorbidities at Diagnosis

At diagnosis, the majority of patients exhibited comorbid conditions. The most prevalent comorbidity was moderate-to-severe valvular heart disease, affecting 83% of patients. Tricuspid regurgitation (TR) is the most prevalent and clinically significant valvular involvement observed in patients with PAH. A majority of them had moderate-to-severe TR, and its severity correlates with disease progression and right heart dysfunction. Pulmonary valve regurgitation is the second most common valvular disease, and in most cases, its degree is moderate. On the other hand, although aortic stenosis (AS) and mitral valve diseases are less common in PAH, but they are still important considerations, especially in cases where left heart dysfunction contributes to elevated pulmonary pressures. The presence of AS or mitral valve disease should be carefully assessed as they can contribute to a mixed picture of pulmonary hypertension and significantly impact the clinical management and prognosis of patients. Patients with underlying severe AS and mitral regurgitation were excluded from this study. This was followed by systemic hypertension in 37% of patients and atrial fibrillation in 20%. A smaller proportion of the patients had ischemic heart disease (12%), and 8% had coronary stents. Overall, 176 of the 194 patients (90.7%) reported at least one comorbidity at diagnosis.

### 3.2. Treatment Patterns

Monotherapy was the most common initial treatment regimen, received by 81.5% of patients. Of these patients, 61.4% from the total group remained on monotherapy, while 20.1% progressed from monotherapy to dual therapy during the observation period. The remaining 18.5% of patients started on dual therapy from the beginning. Among those receiving monotherapy, endothelin receptor antagonists (ERAs) were the most frequently used drugs (72% of cases), while phosphodiesterase-5 inhibitors (PDE5is) were the most common agents in dual therapy (56%). The mean duration of monotherapy was 2.2 years, while dual therapy averaged 3.9 years.

### 3.3. Disease Progression

At both the initial and last visit, 92.1% and 88.5%, respectively, were classified as WHO-FC III, indicating severe PAH. Few patients transitioned across functional class categories, with most transitions occurring from WHO-FC III to WHO-FC IV, signaling a worsening clinical status. The mean mPAP at the last visit was 57 mmHg, suggesting persistent severe disease. The correlation between functional class at the last visit and mortality was significant but weak (coefficient = 0.21, *p*=0.019). Patients, who were presented with significant symptoms, experienced a worsening of their condition, leading to functional status worsening (e.g., moving toward Class IV). Many patients with PAH had comorbid conditions, including right heart failure, chronic kidney disease, or other cardiovascular or respiratory diseases, which can worsen functional class. The presence of these comorbidities might influence the progression of PAH and the functional class of the patient, even if the PAH itself is being managed. The population of this study contains only 2 patients who were identified as FC Class II at the first or last evaluation. As a result of this small sample size, statistical correlations were not computed.

### 3.4. OS

Vital status data were available for 182 patients, of whom, 101 (55%) had died during the observation period. Patients were divided into two subgroups based on treatment compliance: 126 (69.2%) patients were compliant with their treatment regimen, while 56 (30.8%) were classified as lost to follow-up. The characteristics of these two subgroups are shown in Table 2. The compliant group had a younger mean age compared to the lost-to-follow-up group, but otherwise, the two groups were similar in terms of gender distribution, mPAP, CO, 6MWD, and PAH subtype.

Based on the established treatment protocols in the country, follow-up visits are recommended at 6 months period. Follow-up at shorter intervals (3 months) was performed in specific cases where the need for rehospitalization in PH patients was influenced by factors such as disease progression, side effects of therapy, or comorbidities. Common reasons for rehospitalization include right heart failure, worsening symptoms, or rhythm conduction disorders. Analysis of the reasons for therapy discontinuation was out of scope, as available data on this are scarce.

**Figure 1:**
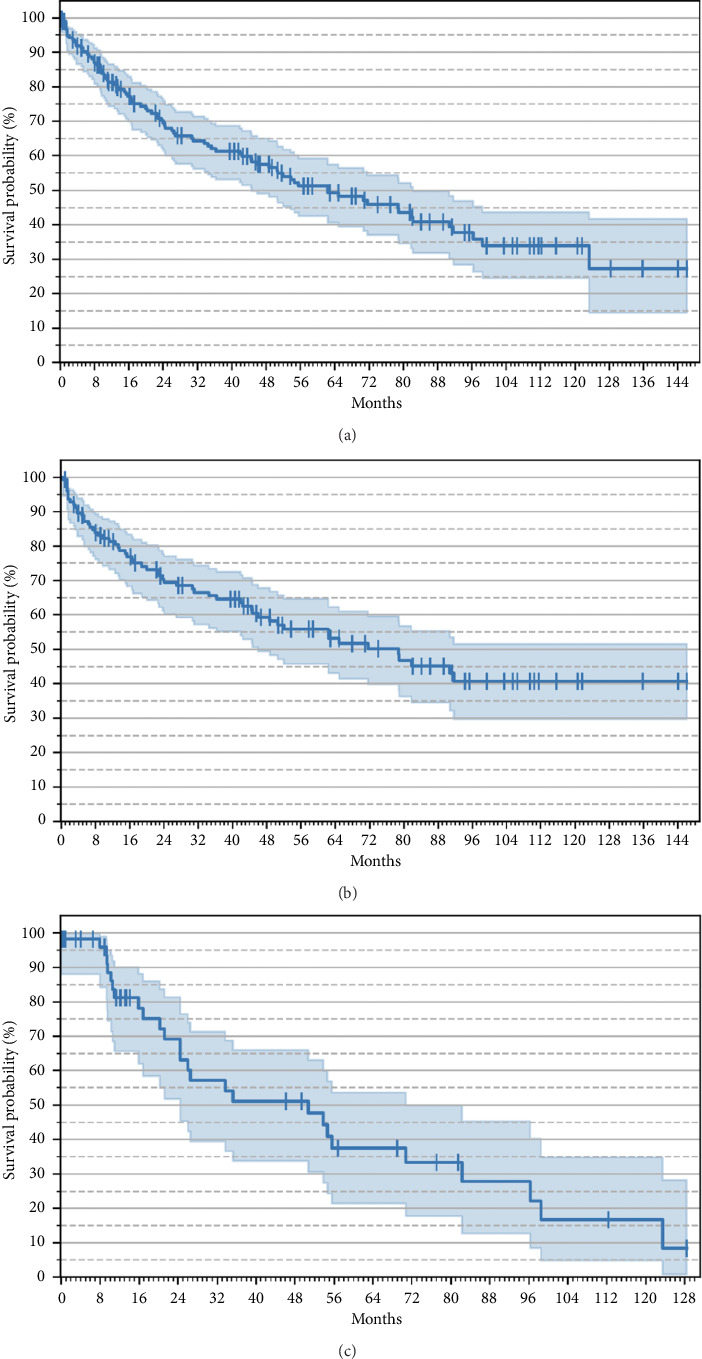
Overall survival. (a) Kaplan–Meier curve in all patients. (b) Kaplan–Meier curve in compliant patients. (c) Kaplan–Meier curve in lost-to-follow-up patients.

OS for the entire cohort and by compliance status is presented in Figure 1. The median OS for the entire cohort was 62.5 months (95% CI: 44.7–82.3 months). The median OS for the compliant group was 78.9 months (95% CI: 46.0-NA), while for the lost-to-follow-up group, the median OS was 50.8 months (95% CI: 24.5–70.8 months).

Additional correlation analysis revealed significant associations between vital status and both age (coefficient = 0.22, *p*=0.002) and 6MWD (coefficient = −0.347, *p* < 0.001). No significant correlation was found between vital status and gender (*p*=0.052), ICD-10 code (*p*=0.493), mPAP at the first visit (*p*=0.5), or CO at the first visit (*p*=0.09).

## 4. Discussion

This study analyzes the epidemiological, clinical, and therapeutic characteristics of PAH patients treated at a major reference center in Bulgaria, highlighting the challenges of delayed diagnosis, high monotherapy rates, and the significant impact of noncompliance on survival.

The demographic characteristics of the patients in this study align with those typically seen in contemporary PH centers. However, one notable difference between our cohort and data reported from Europe and the United States of America is the disproportionately low number of patients diagnosed at earlier stages of the disease (WHO-FC I and II) [8–11]. In our cohort, the majority of patients presented with advanced disease at their first visit, likely due to delayed diagnosis. This delay may contribute to worse clinical outcomes and reflects the need for better screening and earlier referral to specialized centers.

Echocardiography was used to assess right ventricular function and estimate pulmonary artery pressures (PAPs). Elevated PAP indicates PH, and serial echoes can track its progression. In certain follow-up visits, RHC, a more invasive procedure, was chosen not to be performed, and echocardiography served as an excellent alternative to assess the patient's hemodynamic status. RHC is the gold standard for diagnosing pulmonary hypertension and assessing CO, but due to its invasive nature and risks, echo-based measurements are often preferred for regular monitoring or follow-up visits. Our follow-up protocol for patients with PAH consists of a clinical examination every 6 months, which includes echocardiography, 6MWT, and NT-proBNP monitoring. RHC is performed every 12 months if there is no progression of the disease according to the local treatment protocol.

The lack of functional class improvement over the course of treatment is concerning, as it suggests a suboptimal therapeutic response, particularly in the absence of parenteral therapy, which has been shown to improve outcomes in advanced cases [12]. In addition, the slow adoption of guideline-recommended therapies during the study period likely contributed to the limited therapeutic success in this population.

The relatively high proportion of patients remaining on monotherapy (61.4%) in this cohort contrasts with findings from the European PH registry, where combination therapies are more commonly initiated [8–10]. Given that combination therapy is recommended by ERS/ESC guidelines for patients in WHO-FC III or IV to target multiple pathways, the high reliance on monotherapy could reflect challenges in the practical implementation of ERS/ESC recommendations among treating physicians and an opportunity for improved treatment protocols [2]. In our study, ERAs were the most frequently prescribed monotherapy agents (72%), whereas PDE5i was the predominant choice in dual therapy (56%). Notably, no patients in our cohort received triple therapy at the start of treatment, a factor that could have influenced long-term survival outcomes [13]. The absence of triple therapy, which is increasingly being adopted in other countries, likely reflects Bulgaria's slower uptake of innovative therapies.

Despite these therapeutic limitations, the median OS in our cohort, as estimated via the Kaplan–Meier analysis, was 62.5 months. This figure compares favorably with historical data, particularly the prespecific treatment era when the median survival was estimated at 33.6 months (95% Cl: 1.9–3.7 years) [14]. However, a concerning finding in our study is the high rate of noncompliance, with 31% of patients classified as lost to follow-up. Although the reasons for noncompliance were not explicitly captured in the study, factors such as financial barriers, inadequate patient education, follow-up issues, or systemic healthcare limitations likely contributed to the high rate of patients lost to follow-up. This is a critical area for future research, as improving adherence could substantially boost survival and clinical outcomes.

Patients who were compliant with their treatment exhibited a significantly longer median OS (78.9 months) compared to those who were lost to follow-up or untreated (50.8 months). This 28-month difference underscores the potential of continued therapy, even in the absence of newer treatments such as parenteral therapy or lung transplantation. The survival of noncompliant patients in our cohort was still higher than that of historical comparators, highlighting the efficacy of PAH–specific therapy even when adherence is suboptimal. The findings of this study suggest that, while Bulgaria's slower adoption of innovative PAH therapies and the absence of advanced treatment options such as parenteral therapies may worsen outcomes, improving patient compliance could lead to substantial benefits in survival.

This study has several limitations. First, as an observational retrospective study, the influence of additional factors not reflected in the medical records cannot be excluded. For instance, lifestyle factors, socioeconomic status, and other comorbidities may have played a role in patient outcomes but were not accounted for in the analysis. Second, as St. Anna Hospital treats the majority of PAH patients diagnosed in Bulgaria, selection bias is not expected to significantly influence the generalizability of the conclusions for the PAH population in the country. However, missing data are potential limitations. Some patient data, particularly epicrises from half-annual checkups, were stored in paper format before 2018, which may have introduced gaps in the dataset.

## 5. Conclusion

The study demonstrates that while PAH patient demographics in Bulgaria are comparable to those in Europe and the United States of America, treatment patterns differ, with a higher reliance on monotherapy, which appears insufficient for optimal outcomes. Compliant patients showed significantly longer survival, highlighting the importance of adherence to therapy. Access to novel therapies and improved treatment protocols could further enhance outcomes. Establishing a national PAH registry and increasing disease awareness could improve diagnostic rates and enable earlier intervention.

## Figures and Tables

**Table 1:** Patient characteristics at baseline.

Characteristic	Patients (%)	Mean ± SD
Gender, female	135 (69.5%)	—
Age (years)	—	52 ± 15
WHO-FC		—
II	2 (1.0%)	
III	174 (92.1%)	
IV	13 (6.9%)	
mPAP (mmHg)	—	53.5 ± 20.6
CO (L/min)	—	4.3 ± 1.6
TAPSE (mm)	—	17 ± 4.3
PVR (Woods' units)	—	20.4 ± 11.4
RAp (mmHg)	—	12.6 ± 7.6
6MWT distance (m)	—	310 ± 126
BNP (x ULN)	—	9.0 ± 18.6
NT-proBNP (x ULN)	—	4.3 ± 1.1

Abbreviations: 6MWT, 6-min walk test; BNP, brain natriuretic peptide; CO, cardiac output; mPAP, mean pulmonary arterial pressure; NT-proBNP, N-terminal probrain natriuretic peptide; PVR, pulmonary vascular resistance; RAp, right atrial pressure; TAPSE, tricuspid annular plane systolic excursion; ULN, upper limit of normal; WHO-FC, World Health Organization Functional Class.

**Table 2:** Patient characteristics by compliance status.

Characteristic	Compliant group (*n* = 126)	Lost to follow-up group (*n* = 56)
Gender, female (%)	87 (69.0%)	40 (71.4%)
Age (years)	50	59
PAH subgroups		
IPAH	79 (62.7%)	34 (60.7%)
Eisenmenger	24 (19%)	15 (26.8%)
CTD	23 (18.3%)	7 (12.5%)
mPAP (mmHg)	57.6	60.9
CO (L/min)	4.2	4.4
6MWD (m)	312	297

Abbreviations: 6MWD, 6-min walk distance; CO, cardiac output; CTD, connective tissue disease; IPAH, idiopathic pulmonary arterial hypertension; mPAP, mean pulmonary arterial pressure; PAH, pulmonary arterial hypertension.

## Data Availability

Data are available on request from the authors.
